# Dizziness and Driving From a Patient Perspective

**DOI:** 10.3389/fneur.2021.693963

**Published:** 2021-07-01

**Authors:** Roeland B. van Leeuwen, Tjard R. Schermer, Carla Colijn, Tjasse D. Bruintjes

**Affiliations:** ^1^Department of Neurology Gelre Hospitals, Apeldoorn Dizziness Centre, Gelre Hospitals, Apeldoorn, Netherlands; ^2^Department of Epidemiology and Statistics, Apeldoorn Dizziness Centre, Gelre Hospitals, Apeldoorn, Netherlands; ^3^Apeldoorn Dizziness Centre, Gelre Hospitals, Apeldoorn, Netherlands; ^4^Department of Otorhinolaryngology, Apeldoorn Dizziness Centre, Gelre Hospitals, Apeldoorn, Netherlands; ^5^Department of Otorhinolaryngology, Leiden University Medical Center, Leiden, Netherlands

**Keywords:** dizziness, driving, patient perspective, fitness to drive, vertigo

## Abstract

**Background:** People with dizziness may experience driving-related limitations. Few data are available about the impact of dizziness on driving.

**Aim:** The aim of this study is to investigate the impact of dizziness on driving, factors related to impairment (age, gender, and type of diagnosis), and the potential consequences for patients' ability to work. We also investigated whether the patients expected and actually received information about their dizziness-related fitness to drive from their physician.

**Methods:** A cross-sectional, observational study was conducted in the Apeldoorn Dizziness Centre, a tertiary care referral centre for patients with dizziness. A consecutive cohort of patients was asked to complete a study-specific questionnaire about driving.

**Results:** Between January 1, 2020, and December 20, 2020, 432 patients were included. Fifty-six percent of the patients in this group were female. The average age of patients was 58.3 years (SD 16). Overall, 191 of the 432 patients (44%) experienced limitations related to driving, and 40% of the patients who experienced limitations also experienced limitations to work related to their inability to drive. The subject of fitness to drive had not been discussed with their physician in 92% of the patients, and 24% of the whole patient group indicated that they would have liked to discuss this topic. The following factors, independently from each other, increased the chance of experiencing driving-related limitations: younger age, female sex, and the diagnosis of Meniere's disease.

**Conclusion:** Dizzy patients, especially younger patients, women, and patients with Meniere's disease, regularly experience limitations related to driving, and this often means that they are unable to work. Driving is hardly ever discussed during a medical consultation. In our opinion, the topic of driving and dizziness should always be addressed during medical consultations in dizzy patients.

## Introduction

Dizziness is a common presenting complaint in patients, both in primary care and in the hospital. Dizziness and vertigo may be associated with significant morbidity and may have a profound impact on the quality of life, especially in the older population (1) This means that it is important to establish an accurate diagnosis and treatment plan quickly, even though dizziness and vertigo often has a benign origin.

Few data are available about the impact of dizziness on driving in patients with dizziness (2–4). At the same time, the (in)ability to drive can have a large impact on the quality of life of these patients (5). A number of factors play a role in deciding whether a patient with dizziness and vertigo should be allowed to drive. What does the national law say about driving restrictions? Are these restrictions known by the health professionals and/or the patients? Are patients informed by health professionals about the potential restrictions? Which considerations play a role in the patient's decision whether or not to drive?

As far as we know, the question how patients with dizziness deal with driving has only been investigated in three previous studies (2–4). Because we wanted to know what percentage of dizzy patients experience inability to drive, we performed an observational study in this patient group. The research questions focused on the frequency of impairment to driving in patients with dizziness, factors related to impairment (age, gender and type of diagnosis), and the potential consequences for the patients' ability to work. Furthermore, we wanted to investigate whether the patients expected and actually received information about fitness to drive from their physician.

## Methods

A cross-sectional, observational study was conducted in the Apeldoorn Dizziness Centre—a tertiary care referral centre for patients with dizziness as their main complaint. We used the term “dizziness” to describe the complaint from a patient perspective. The terms “dizziness and vertigo” were used as an umbrella term to describe the symptoms from a doctor's point of view (6).

In our centre, all patients routinely complete several questionnaires and undergo a physical examination and advanced audiovestibular testing. During a joint consultation with an ENT surgeon and a neurologist, the diagnosis is made in consensus between the two specialties. In the period between January 1, 2020, and December 20, 2020, a consecutive cohort of patient was asked to complete a study-specific questionnaire about driving. Only those patients who used to drive before their dizziness complaints started were included in the study. The questions that were asked are set out in Table 1. The entire patient sample was asked to respond to questions 1, 6, and 7. Questions 2, 3, 4, and 5 were only applicable for patients who experienced limitations related to driving. For all patients, the following data were collected from their medical records: sex, age, primary diagnosis, and (if applicable) a second diagnosis.

**Table 1:** Answers to questionnaire about limitations in driving in 432 dizziness patients of the tertiary care referral center.

	**Questionnaire**	***n***	
1	Did you experience driving-related limitations as a result of your dizziness?	432	Yes: 44.2%
			No: 55.8%
2	How many periods have you experienced in which you were unable to drive?	185	1–5 periods: 60%
			6–10 periods: 11%
			>10 periods: 29%
3	For how long have you had dizziness complaints?	190	1–6 months: 25%,
			6–12 months: 16%
			>12 months: 59%
4	What was the length of the longest period in which you were unable to drive?	184	1–6 days: 30%
			1–4 weeks: 26%
			1–3 months: 23%
			>3 months: 21%
5	Were you unable to work as a result of the inability to drive?	173	Yes: 40%
			No: 60%
6	Did your doctor inform you about potential restrictions on driving?	406	Yes: 8%
			No: 92%
7	Did you miss receiving information about dizziness and driving?	405	Yes: 24%
			No: 76%

The local ethics committee of Gelre hospitals authorized the study and patients provided written informed consent for the use of information from their medical records and the completed questionnaires for the study.

### Statistical Analysis

Demographic characteristics and diagnoses underlying the dizziness and vertigo were compared between two subgroups of patients (i.e., those with and those without driving limitations) using Student's *t*-test (for age) and Chi-square tests (for sex and diagnoses). Multivariate logistic regression analysis was performed to calculate odds ratios (ORs) for experiencing limitations due to dizziness related to driving as the dichotomous dependent variable and the patient's age, sex, and diagnoses underlying the dizziness as the independent variables. We categorized the patients in various age groups. Only diagnoses with a prevalence in the study population of at least five patients with and five patients without limitations due to dizziness while driving were included in the logistic regression model (i.e., vestibular migraine, unknown unilateral vestibular hypofunction, bilateral vestibular hypofunction, vestibular paroxysmia, Meniere's disease, vestibular neuritis, BPPV, and anxiety disorder/hyperventilation syndrome). Using likelihood ratio-based backward stepwise elimination of variables, the initial full model was reduced to a final model. Goodness of fit of the logistic regression models was assessed using Hosmer and Lemeshow test. All analyses were performed using SPSS (IBM SPSS Statistics for Windows, Version 25.0. Armonk, NY: IBM Corp.). *p* < 0.05 were considered statistically significant in all analyses.

## Results

Overall, 432 patients were included in the study. Fifty-six percent of the patients in this group was female. The average age of patients was 58.3 years (SD 16).

The most frequent diagnoses were hyperventilation/anxiety (34%), benign paroxysmal positional vertigo (BPPV, 22%), Meniere's disease (7%), vestibular migraine (6%), vestibular neuritis (5%), vestibular paroxysm (4%), bilateral vestibular hypofunction (3%), and benign recurrent vertigo (2%). No diagnosis could be made in 13% of the patients. A second diagnosis was made in 22.5% of the patients.

The answers to the questions in the questionnaire are set out in Table 1. Overall, 191 of the 432 patients (44%) experienced limitations related to drive, and 40% of the patients who experienced limitations also experienced limitations to work related to their inability to drive. The subject of driving had not been discussed with their physician in 92% of the patients, and 24% of the whole patient group indicated that they would have liked to discuss this topic.

The data of the groups without and with limitations are presented in Table 2.

**Table 2:** Characteristics of the total study population and the subgroups with and without limitations in driving.

**Variables**	**Total group (*n* = 432)**	**Group without limitations (*n* = 241)**	**Group with limitations (*n* = 191)**	***p*-value**
Female (%)	243 (56)	116 (48)	127 (67)	<0.001
Age in years (mean, SD)	58.3 (16)	62.6 (16)	52.9 (15)	<0.001
Age, in categories (%)				<0.001
≤ 40 years	68 (16)	26 (11)	42 (22)	
41–50 years	69 (16)	23 (10)	46 (24)	
51–60 years	83 (19)	41 (17)	42 (22)	
61–70 years	87 (20)	59 (24)	28 (15)	
≥71 years	125 (29)	92 (38)	33 (17)	
Meniere's disease (%)	29 (7)	10 (4)	19 (10)	0.020
Vestibular migraine (%)	26 (6)	9 (4)	17 9)	0.040
BPPV (%)	93 (22)	53 (22)	40 (21)	0.815
Hyperventilation/anxiety (%)	149 (34)	75 (31)	74 (39)	<0.001
Unknown unilateral vestibular hypofunction (%)	20 (5)	7 (4)	13 (3)	0.066
Benign recurrent vertigo (%)	9 (2)	7 (3)	2 (1)	0.310
Bilateral vestibular hypofunction (%)	13 (3)	6 (3)	7 (4)	0.575
Vestibular paroxysmia (%)	15 (4)	9 (4)	6 (3)	0.797
Vestibular neuritis (%)	23 (5)	13 (5)	10 (5)	1.000

*BPPV, Benign paroxysmal positional vertigo; SD, standard deviation*.

The following factors, independently from each other, influenced the chance of experiencing driving limitations: age [compared to patients aged ≥71 years, the three subgroups below the age of 60 all showed statistically significant ORs > 3; being 1 year older reduced the risk (i.e., OR = 0.96; 95% CI 0.95 to 0.98)], sex {being a female increased the risk [OR = 1.99 (95%CI 1.30 to 3.05)]}, and Meniere's disease {being diagnosed with Meniere's disease increased the risk [OR = 3.90 (95%CI 1.70 to 8.94) compared to other diagnoses underlying the dizziness; see Table 3]}.

**Table 3:** Results of the multivariate logistic regression model for associations between experiencing driving limitations due to dizziness and vertigo and patient demographic characteristics and underlying diagnoses (*n* = 432).

	**Reference**	**OR**	**95% CI**	***p*-value**
**Full model**				
Female	Male	1.92	1.24, 2.97	0.004
Age categories				
≤ 40 years	≥71 years	4.70	2.34, 9.44	<0.001
41–50 years	≥71 years	6.52	3.22, 13.20	<0.001
51–60 years	≥71 years	3.15	1.67, 5.94	<0.001
61–70 years	≥71 years	1.28	0.68, 2.41	0.441
Meniere's disease	No Meniere's disease	4.99	2.08, 11.95	<0.001
Vestibular migraine (VM)	No VM	1.90	0.83, 4.38	0.131
BPPV	No BPPV	1.65	0.94, 2.89	0.078
Anxiety disorder	No anxiety disorder	1.84	0.83, 4.06	0.131
Hyperventilation	No hyperventilation	0.99	0.61, 1.63	0.977
Unknown unilateral vestibular hypofunction (UUVH)	No UUVH	0.88	0.34, 2.28	0.796
Bilateral vestibular hypofunction (BVH)	No BVH	2.38	0.80, 7.10	0.121
Vestibular paroxysmia (VP)	No VP	1.84	0.61, 5.49	0.277
Vestibular neuritis (VN)	No VN	1.24	0.49, 3.11	0.654
Reduced model^*^				
Age categories				
≤ 40 years	≥71 years	4.33	2.26, 8.29	<0.001
41–50 years	≥71 years	5.51	2.86, 10.63	<0.001
51–60 years	≥71 years	2.89	1.58, 5.30	0.001
61–70 years	≥71 years	1.32	0.71, 2.45	0.378
Female	Male	1.99	1.30, 3.05	0.002
Meniere's disease	No Meniere's disease	3.90	1.70, 8.94	0.001

*CI, confidence interval; OR, odds ratio*.

* *Using backward stepwise elimination of variables from the full model*.

## Discussion

Our study shows that a rather high percentage of patients with dizziness and vertigo (44%), who have been seen in a tertiary care centre, experience limitations related to driving. Limitations were more frequent among younger patients, women, and patients diagnosed with Meniere's disease. In only a few cases had the subject of driving been discussed with the health professional.

It is not clear why younger patients experience more limitations than elderly patients. It is possible that younger patients use their car more frequently than elderly patients for work, leisure, or both. Furthermore, they might make more use of the motorway. We cannot explain why women experience more limitations while driving. Perhaps, because women generally drive more carefully than men, dizzy women may experience more limitations in driving than men (7). The percentage of patients with driving limitations in our study is higher than found in previous studies (14–35%) (2–4). It should be noted that in one of these studies, only patients with Meniere's disease were included (4). The higher prevalence of experiencing driving-related limitations among patients with Meniere's disease confirms the findings reported by Cohen et al. (3).

In 40% of the patients with limitations related to driving, this resulted in absence from work. Therefore, the inability to drive has major consequences for a large group of patients with dizziness and vertigo. We have been unable to find data in other published studies on the impact of not being able to drive for the patient's ability to work.

In only 8% of the patients, the topic of fitness to drive had been discussed with the patient's physician. Sindwani's study shows that 10% of dizzy patients received a negative advice about driving (2). While a significant number of patients in our study indicated that they would have liked more clarity on this, and while the Dutch government assumes that health professionals are under an obligation to inform patients about potential limitations related to driving, in 92% of the patients with dizziness and vertigo in our study, the topic of driving was not discussed. This could be because doctors are not aware of the requirements imposed by national law (8, 9). Another explanation could be that doctors in the Netherlands regard the current legislation as too strict. As a result, they might decide not to discuss this topic with their patients. Previous studies have shown that many patients (50–60%) do not intend to comply with the imposed restrictions on driving (2, 3).

A limitation of our study may be that it was conducted in a tertiary centre. This means that we see a selection of patients with often more severe complaints. Moreover, our study does not show why patients with dizziness and vertigo often experience the inability to drive. This could be because of physical complaints or because of insecurity or anxiety. Our study did not aim at getting more clarity about these or other potential explanations. As to the question about inability to work, we do not know how many patients were in fact employed or self-employed.

Further research on the reasons for the limitations with respect to driving and the possibility of dealing with these limitations through advice or treatment is warranted.

In conclusion, dizzy patients, especially younger patients, women, and patients with Meniere's disease, regularly experience limitations related to driving, and this often means that they are unable to work. Driving is hardly ever discussed during the medical consultation. As such, doctors should always discuss the topic of driving and dizziness as part of their management of dizzy patients.

## Data Availability Statement

The original contributions presented in the study are included in the article/supplementary material, further inquiries can be directed to the corresponding author/s.

## Author Contributions

RL, TS, and TB contributed to conception and design of the study. CC organized the database. TS performed the statistical analysis. RL wrote the first draft of the manuscript. TS, CC, and TB wrote sections of the manuscript. All authors contributed to manuscript revision, read, and approved the submitted version.

## Conflict of Interest

The authors declare that the research was conducted in the absence of any commercial or financial relationships that could be construed as a potential conflict of interest.
